# Caregiving Through Turbulent Times: Findings From the MIT AgeLab’s Longitudinal Study of Family Caregivers

**DOI:** 10.1093/geroni/igab046.697

**Published:** 2021-12-17

**Authors:** Julie Miller, Julie Miller

## Abstract

Nearly one in five Americans is an unpaid family caregiver, and the need for family caregivers is projected to grow over the next several decades in the face of longer lifespans (AARP 2020). Yet the increasing centrality of family caregivers for providing care to an aging population highlights two knowledge gaps: first, the degree and experience of burden and stress caregivers manage around balancing care with other family and work responsibilities; and second, a lack of knowledge about the caregiver journey and the microtasks of care, including how caregivers leverage – or not – different tools, technologies and resources to support the care they provide. To develop a deeper understanding of these questions and others, the MIT AgeLab has built a research panel of over 1200 caregivers providing care to another adult family member. This symposium will present findings from the MIT AgeLab Caregiver Panel, including: 1) an examination of the extent to which family caregivers identify as such and how they feel about their roles; 2) how family caregivers experienced the COVID-19 pandemic both personally and around the care they provide; 3) caregivers’ use of and attitudes toward technology to support the care they provide; and 4) what caregivers identify as their key unmet needs. The session will include a facilitated discussion around the intersection of COVID-19 with caregivers’ technology use, experience of caregiving, and future needs, as well as to identify additional research questions and directions for future research with the MIT AgeLab Caregiver Panel.

